# Carotid Artery Intima Media Thickness and HsCRP; Predictors for Atherosclerosis in Prediabetic Patients?

**DOI:** 10.12669/pjms.292.3133

**Published:** 2013-04

**Authors:** Hulya Parildar, Oyku Gulmez, Ozlem Cigerli, Asli Dogruk Unal, Rengin Erdal, Nilgun Guvener Demirag

**Affiliations:** 1 Hulya Parildar, MD, Department of Family Medicine, Baskent University Istanbul Hospital, Istanbul, Turkey.; 2 Oyku Gulmez, MD, Department of Cardiology, Baskent University Istanbul Hospital, Istanbul, Turkey.; 3 Ozlem Cigerli, MD, Department of Family Medicine, Baskent University Istanbul Hospital, Istanbul, Turkey.; 4 Asli Dogruk Unal, MD, Department of Endocrinology and Metabolism, Baskent University Istanbul Hospital, Istanbul, Turkey.; 5 Rengin Erdal. MD, Department of Family Medicine, Baskent University, Ankara, Turkey.; 6 Nilgun Guvener Demirag, MD, Department of Endocrinology and Metabolism, Baskent University Istanbul Hospital, Istanbul, Turkey.

**Keywords:** Prediabetes, Carotid atherosclerosis, Inflammation

## Abstract

***Objective:*** We aimed to assess carotid intima media thickness (CIMT) and serum high-sensitivity C-reactive protein (hs-CRP) levels as estimated markers of subclinical atherosclerosis and inflammation in prediabetic patients.

***Methodology:*** One hundred and ten patients were defined as prediabetic and seventy-six subjects (age and sex matched) were assigned as control group in our cross sectional study. Bilateral CIMT measurements and hs-CRP levels were evaluated.

***Results: ***The prevalance of hypertension, hyperlipidemia, angiotensin receptor blockers and antihyperlipidemic medication use were statistically higher in the prediabetic group. Serum hs-CRP levels, left, right and maximum CIMT were statistically higher among prediabetics compared to control group. There was a positive, significant correlation between left, right, maximum CIMT and fasting blood glucose, HbA1c, hs-CRP levels and BMI.

***Conclusion:*** Recognising and focusing on the intervention of prediabetic state as early as possible and identifying the susceptible patients who may benefit from more aggressive preventive therapy is an important issue of primary prevention of diabetes and cardiovascular diseases.

## INTRODUCTION

Cardiovascular (CV) disease is still the leading cause of mortality in many countries.^1^ The prevalence of its risk factors especially obesity and diabetes, continues to increase at an alarming level.^2^ Hyperglycemia, insulin resistance and CV disease have been associated with chronic and subclinical inflammation, as indicated by elevated circulating levels of proinflammatory proteins.^3^^-^^5^ Among the markers of inflammation, hs-CRP is the most studied, with evidence that it may also play a direct role in atherosclerotic lesion formation.^6^ For those individuals especially for those at intermediate risk for cardiovascular diseases according to global risk scores, other markers like high sensitive C-reactive protein (hs-CRP), and carotid intima-media thickness (CIMT) may be useful in predicting the individuals who may benefit from more aggressive preventive therapy.^7^ CIMT is considered as a surrogate marker of cardiovascular disease, an independent risk factor, and a tool for early detection of atherosclerosis and also has the ability to determine the management strategy.^8^^-^^10^

The main objective of the study was to assess CIMT and hs-CRP levels as estimated markers of subclinical atherosclerosis and inflammation in prediabetic patients with no history of cardiovascular disease.

## METHODOLOGY

This descriptive, cross-sectional study included 110 prediabetic patients as case, 76 healthy subjects as control group. Clinical history, current medication use, and risk factors for CV disease were recorded. The study was approved by the local Ethics Committee of Baskent University Hospital in Ankara, Turkey (N:KAO8/129). The main inclusion criteria were no personal history of cardiovascular disease, no premature cardiovascular disease in the first-degree relatives, and serious illness requiring admission to hospital in the past year. Excluded were individuals with an underlying inflammatory disease, any infection or other inflammatory condition, including infarction, surgery or angiography during the 6 months prior to enrollement, and those being treated with steroid or non-steroidal anti-inflammatory medication except for aspirin at doses lower than 325 mg per day.

 Prediabetic state was determined according to World Health Organisation (WHO) criteria based on fasting serum 2h glucose levels by a 75 gr standart oral glucose tolerance test. Hypertension was defined as blood pressure of ≥140/90 mmHg or use of anti-hypertensive drugs. Hyperlipidemia was defined as a LDL-chol level of ≥130 mg/dl, and/or triglyceride level of ≥150 mg/dl, HDL-chol level of <45mg/dl or statin and/or fibrate use.

Measurement of CIMT was performed in the posterior wall of both carotid arteries by mode B ultrasound with an Acuson Sequoia ultrasonography device equipped with a lineer probe operating 8 mHz. Maximum CIMT was calculated. All scans were conducted by an experienced cardiologist, who had no prior knowledge of the patient's clinical characteristics.

Highly sensitive levels of hs-CRP were measured by an immunoturbidimetric assay (C8000 system) with normal levels as <0-5 mg/dl. Blood glucose levels were measured by enzymatic colorimetric assay (C8000 system). Insulin levels were analysed with CMIA (Architect i1000 system, Abbott, USA), NA: 2.6-24.9μU/ml. HbA1c was detected with a turbidimetric assay method (C4000, Architect cSystem). Serum total cholesterol, triglyceride, HDL-chol, LDL-chol were measured with enzymatic colorimetric assay (C8000 system, Abbott, USA).

Statistical analysis was performed using SPSS 16.0 for Windows. Student T test and Pearson correlation test were used for statistical comparisons. Mann–Whitney U test was used for nonparametric data. Stepwise linear regression models were used to study the association between CIMT after adjusting for basic covariates. Statistical significance was set at p value of less than 0.05.

## RESULTS

One hundred and ten patients defined as prediabetic and seventy-six healthy subjects were assigned as case and control group, respectively. Mean (SD) age of prediabetic patients was 51.1±9.9 years, the percentage of female patients was 68.1% (n=75). There were no significant differences between the groups in terms of age, gender and smoking rates. BMI value, the prevalance of hypertension, hyperlipidemia, angiotensin receptor blockers (ACEI and/or ARB) and antihyperlipidemic medication use were found to be statistically higher in prediabetic group (Table-I).

Among biochemical parameters, serum insulin, fasting blood glucose and HbA1c, hs-CRP levels were significantly higher, serum triglyceride levels were significantly lower in prediabetic group due to antihyperlipidemic medication use (Table-II).

Left, right and maximum CIMT were statistically higher among prediabetics (Table-III). Stepwise regression analysis was carried out among Hs-CRP, serum blood glucose, age and BMI variables in order to predict the variability of maximum CIMT. According to this model, age and BMI were found to be responsible for 40% of the variability for maximum CIMT (Table-IV). Maximum CIMT values were not significantly different regarding the gender of the subjects. Adjustment for other covariables, age and BMI were associated with CIMT in both gender. There was a positive, significant correlation between left, right, maximum CIMT and fasting blood glucose, HbA1c levels. There was a positive, significant correlation between left, right, maximum CIMT, hs-CRP and BMI (Table-V).

**Table-I:** Patient demographics, comorbities, rates of tobacco and medication use

	*Control Group* *(n=76)*	*Prediabetic Group (n=110)*	*P*
Age (mean±sd)	50.1±7.8	51.1±9.9	0.54
Gender (F/M, n)	55/21	75/35	0.62
BMI (kg/m^2^)	26.2±4.6	30.3±5.7	<0.001
Hypertension (n)	8	48	<0.001
Hyperlipidemia (n)	20	59	<0.001
ACEI/ARB use (n)	6	42	<0.001
Statin/fibrate use (n)	9	44	<0.001
Tobacco smoking(n)	10	19	0.46

**Table-II:** Comparison of biochemical laboratory findings

	*Control Group* *(n=76)*	*Prediabetic Group (n=110)*	*p*
Fasting blood glucose(mg/dl)	93.5±7.9	106.9±8.7	<0.001
Serum fasting insulin (mg/dl)	10.5±5.2	15.1±8.4	0.003
HbA1c (%)	5.1±0.4	5.8±0.4	<0.001
Hs-CRP (mg/dl)	2.3±2.0	3.5±3.2	0.040
Total chol (mg/dl)	213.7±50.6	235.1±22.3	0.530
LDL-chol (mg/dl)	133.0±37.3	154.9±20.1	0.400
HDL-chol (mg/dl)	56.5±35.3	50.4±36.5	0.280
Triglyceride (mg/dl)	134.5±168.4	100.4±89.5	0.030

**Table-III:** The comparison of CIMT values

	*Control Group (n=76)*	*Prediabetic Group* *(n=110)*	*p*
Left CIMT (mm)	0.62±0.16 (0.30-1.0)	0.78±0.23 (0.50-1.70)	<0.001
Right CIMT (mm)	0.63±0.14 (0.40-1.0)	0.73±0.18 (0.40-1.30)	<0.001
Maximum CIMT (mm)	0.62±0.14 (0.35-1.0)	0.75±0.19 (0.45-1.40)	<0.001

**Table-IV:** The results of stepwise linear regression analysis (Model 1, 2) between means of maximum CIMT and basic covariates

*Model*	*R*	*R Square*	*Adjusted R Square*	*Std. Error of the Estimate*	*R Square Change*	*F Change*	*Sig. F Change*
1	0.561^a^	0.315	0.303	1.85729	0.315	25.319	0.000
2	0.655^b^	0.430	0.408	1.71070	0.114	10.830	0.002

**Table-V:** The correlation between CIMT value and other parameters

*Correlations*									
		*Right CIMT (mm)*	*Left CIMT (mm)*	*Maximum CIMT (mm)*	*Serum fasting Insulin (mg/dl)*	*Fasting blood glucose (mg/dl)*	*HbA1c* *(%)*	*BMI (kg/cm* ^2^ *)*	*Hs-CRP (mg/dl)*
Right CIMT (mm)	*r*		0.835^**^	0.947^**^	0.159	0.290^**^	0.244^**^	0.266^**^	0.184^*^
	*p*		0.000	0.000	0.060	0.000	0.008	0.002	0.050
Left CIMT (mm)	*r*			0.968^**^	0.166^*^	0.305^**^	0.301^**^	0.334^**^	0.201^*^
	*p*			0.000	0.049	0.000	0.001	0.000	0.033
Maximum CIMT (mm)	*r*				0.171^*^	0.312^**^	0.288^**^	0.318^**^	0.203^*^
	*p*				0.043	0.000	0.002	0.000	0.031
Serum fasting insulin (mg/dl)	*r*					0.313^**^	0.291^**^	0.451^**^	0.100
	*p*					0.000	0.002	0.000	0.312
Fasting blood glucose (mg/dl)	*r*						0.395^**^	0.313^**^	0.061
	*p*						0.000	0.000	0.520
HbA1c (%)	*r*							0.091	0.049
	*p*							0.389	0.647
BMI (kg/m^2^)	*r*								0.308^**^
	*p*								0.003
Hs-CRP (mg/dl)	*r*								
	*p*								

**. Correlation is significant at the 0.01 level (2-tailed). *. Correlation is significant at the 0.05 level (2-tailed)

## DISCUSSION

Guidelines recommend population based screening algorithms that include Framingham Risk Score, Systematic Coronary Risk Evaluation (SCORE), and Reynolds Score.^11^^-^^13^ However these models are less useful in assessing individual risk. Noninvasive imaging studies and novel biomarkers such as CIMT and hs-CRP have led to an interest at predicting individual risk.

In recent years, CIMT has been shown as an independent predictor of CV risk and the presence of carotid plaque as a strong predictor of CV events and mortality.^14^ These findings highlight the importance of recognizing and managing early stages of atherosclerosis for effective cardiovascular prevention. The American Society of Echocardiography guidelines recommend CIMT assessment in those with FRS of 6-20% without established coronary artery disease, peripheral artery disease, cerebrovascular disease, DM, abdominal aort aneurysm as well as a positive family history of premature CV events, individuals <60 years old with a severe abnormality in any single risk factor, or women <60 years old with at least 2 risk factors for cardiovascular diseases.^15^

Hs-CRP serves as a marker of inflammation and predicts risk of adverse cardiovascular events. Moreover, chronic subclinical inflammation is associated with prediabetic state and a significant lineer increase in incidence of new diabetes with increasing quartiles of hs-CRP.^16^

We found higher CIMT and hs-CRP levels in prediabetic patients compared to control group. Several prospective studies have shown increased CIMT in diabetic patients than in nondiabetic subjects and predicts future events of coronary heart disease.^17^^,^^18^

Several studies reported that traditional CV risk factors are associated with increased CIMT both in diabetic and nondiabetic population.^19^ There was a positive, significant correlation between left, right, maximum CIMT and blood glucose, HbA1c, BMI and hs-CRP (Table-V) in our study, consistent with the previous studies.

In a study, the CIMT values were found higher in obese patients compared to non-obese type 2 diabetic patients without history of coronary artery disease.^20^ In our study CIMT was also positively correlated with BMI. Obesity is an important risk factor for cardiometabolic disorders.

This study had some important limitations which is a cross-sectional study with a relatively small number of subjects, the validity of clinical measures as a screening tool should be verified in studies with larger sample size and cross-sectional study may limit assessing the links. Another limitation was we could not analyse IFG or IGT separately since their numbers were so small. Lastly, the use of medications such as the use of statins, ACEIs and/or ARBs, may cause complex associations in our study with their pleiotropic or anti-inflammatory effects.

We suggest that novel noninvasive techniques and biomarkers such as CIMT and hs CRP may help to determine the individuals who would benefit most from these interventions. Therefore recognizing and focusing on the intervention of prediabetic state and identifying the individuals who are at high risk for cardiovascular events through screens and thus targeting them for individual risk evaluation to start for management as early as possible is very important for the primary prevention of diabetes and cardiovascular diseases.

## Authors Contribution:

Hulya Parildar and Ozlem Cigerli: General design, writing the manuscript, data acquisition, interpretation, final approval of the version.

Oyku Gulmez: General design, data acquisition, interpretation, final approval of the version.

Asli Dogruk Unal: Data acquisition, interpretation, final approval of the version.

Rengin Erdal and Nilgun Guvener Demirag: General design, interpretation, final approval of the version.
